# Enhancing the coverage of SemRep using a relation classification approach

**DOI:** 10.1016/j.jbi.2024.104658

**Published:** 2024-05-21

**Authors:** Shufan Ming, Rui Zhang, Halil Kilicoglu

**Affiliations:** a School of Information Sciences, University of Illinois Urbana-Champaign, 501 E Daniel St., Champaign, 61820, IL, USA; b Division of Computational Health Sciences, Department of Surgery, University of Minnesota, 516 Delaware St SE, Minneapolis, 55455, MN, USA

**Keywords:** Biomedical relation extraction, Relation classification, Large language models, SemRep, SemMedDB

## Abstract

**Objective:**

Relation extraction is an essential task in the field of biomedical literature mining and offers significant benefits for various downstream applications, including database curation, drug repurposing, and literature-based discovery. The broad-coverage natural language processing (NLP) tool SemRep has established a solid baseline for extracting subject–predicate–object triples from biomedical text and has served as the backbone of the Semantic MEDLINE Database (SemMedDB), a PubMed-scale repository of semantic triples. While SemRep achieves reasonable precision (0.69), its recall is relatively low (0.42). In this study, we aimed to enhance SemRep using a relation classification approach, in order to eventually increase the size and the utility of SemMedDB.

**Methods:**

We combined and extended existing SemRep evaluation datasets to generate training data. We leveraged the pre-trained PubMedBERT model, enhancing it through additional contrastive pre-training and fine-tuning. We experimented with three entity representations: mentions, semantic types, and semantic groups. We evaluated the model performance on a portion of the SemRep Gold Standard dataset and compared it to SemRep performance. We also assessed the effect of the model on a larger set of 12K randomly selected PubMed abstracts.

**Results:**

Our results show that the best model yields a precision of 0.62, recall of 0.81, and F_1_ score of 0.70. Assessment on 12K abstracts shows that the model could double the size of SemMedDB, when applied to entire PubMed. We also manually assessed the quality of 506 triples predicted by the model that SemRep had not previously identified, and found that 67% of these triples were correct.

**Conclusion:**

These findings underscore the promise of our model in achieving a more comprehensive coverage of relationships mentioned in biomedical literature, thereby showing its potential in enhancing various downstream applications of biomedical literature mining. Data and code related to this study are available at https://github.com/Michelle-Mings/SemRep_RelationClassification.

## 1. Introduction

The size and growth of biomedical literature presents challenges for biomedical scientists and clinical researchers in keeping up with the latest advancements in their fields and use the knowledge in the literature effectively for discovery and patient care. As of May 2024, PubMed, the primary biomedical bibliographic databases, covers more than 37 million articles, and this number continues to grow with more biomedical literature becoming available online every day. Biomedical relation extraction (RE) aims to automatically extract structured knowledge in the form of entities and their semantic relationships from unstructured biomedical text and is an essential task in biomedical literature mining [1]. Such relationships capture information about the nature and associations between biomedical concepts and thus can support many downstream tasks, i.e., pharmacovigilance [2,3], drug repurposing [4–6], literature-based discovery [7,8], and clinical decision support [9].

While biomedical RE has long been an important target in biomedical text mining, widely-used, accurate tools have remained elusive. Most approaches to biomedical RE are based on supervised machine learning and are trained on corpora that are limited to one or few relationship types [10], which limits their utility. In contrast, SemRep [11, 12], developed at the U.S. National Library of Medicine, is a rare broad-coverage RE tool that extracts subject–predicate–object triples from biomedical text. It is a rule-based system that relies heavily on the UMLS domain knowledge [13] and linguistic principles. SemRep forms the backbone of the Semantic MEDLINE Database (SemMedDB) [14], a PubMed-scale repository of semantic relations extracted from PubMed titles and abstracts. As of February 2024, SemMedDB contains about 129M triples extracted from about 37M publications. For downstream tasks that use biomedical relations broadly and SemMedDB specifically, it is essential that the extracted relations be both accurate and comprehensive. The most complete evaluation of SemRep has shown a precision of 0.69 and recall of 0.42 on a set of 500 sentences from 308 abstracts [12]. While the precision is reasonable, low recall indicates that more than half of the relations expressed in text are being missed. It is imperative to increase SemRep recall, to improve its utility and that of SemMedDB for downstream tasks.

RE is often formulated as a binary or multi-class classification task in a supervised setting. In recent years, Transformer-based language models have been proposed to facilitate biomedical RE using a relation classification approach [10,15–18]. These studies focused one or a limited set of relation types.

In this paper, we investigate whether a relation classification approach can complement SemRep and increase its recall, thereby potentially augmenting the SemMedDB database. To enable model training and evaluation, we curated a dataset from the SemRep Gold Standard data [19], as well as additional datasets in which SemRep triples were evaluated by experts for accuracy [6,20]. In our model, we take named entities that have already been identified by SemRep as input and simply focus on relation classification (i.e., whether a pair of entities in a single sentence are related, and if so, the type of their relation). We train our models by fine-tuning the Transformer-based PubMedBERT model [21]. Drawing inspiration from prior research [17,22], we conducted a series of experiments involving different entity features and model architectures. Our experiments revealed that further pre-training on augmented data using contrastive learning, followed by fine-tuning the model on a task-specific dataset, yielded the highest performance. To understand the complementarity of approaches, we compared this best-performing model with SemRep across 22 categories. To estimate the effect of the model on SemMedDB size and quality, we applied the model to a larger sample of 12K PubMed articles and performed a further manual evaluation of 506 triples.

The main contributions of this study are as follows (see also Table 1):

- We complement the knowledge-based RE tool SemRep by fine-tuning the PubMedBERT model on entities extracted by SemRep, which results in a 44 absolute percentage point increase in overall recall.
- Applying the model to a sample of 12K PubMed articles shows that the model is complementary to SemRep and could potentially double the size of the SemMedDB.
- For some important causal/mechanistic relations, such affects, augments, predisposes, and causes, the model demonstrated a notable improvement, increasing the recall by a factor of two.
- A manual evaluation of 506 triples identified only by the model shows that 67% of them are correct, suggesting that the model is able to maintain SemRep precision.

## 2. Related work

### 2.1. SemRep

SemRep extracts semantic relations in the form of subject–predicate–object triples from biomedical text [11,12]. It follows a pipeline architecture, combining domain knowledge in the UMLS with syntactic and semantic rules. The initial *pre-linguistic analysis* component consists of sentence splitting, tokenization, and acronym/abbreviation resolution steps. This is followed by *lexical/syntactic analysis*, which incorporates lexical lookup to UMLS SPECIALIST Lexicon, part-of-speech tagging, and shallow parsing. Next, *referential analysis* uses MetaMap [23] to map noun phrases to UMLS Metathesaurus concepts and dictionary lookup to map gene/protein names to NCBI Gene identifiers. Finally, *relational analysis* uses indicator rules and syntactic and semantic constraints to extract subject–predicate–object triples, where subject and object are UMLS Metathesaurus or NCBI Gene concepts, and the predicate is a UMLS Semantic Network relation. SemRep optionally performs anaphora resolution [24], extending the scope of relations beyond single sentences. For more detailed information on SemRep, we refer the reader to the overview paper [12].

For illustration, consider the sentence in (1) taken from a PubMed abstract (PMID: 16109976). Semantic triples extracted by SemRep are shown in (2). Relation arguments (subject and object) are represented as CUI: Concept Name (Semantic Type), where CUI is the UMLS Metathesaurus concept unique identifier.

In preterm lambs, intraamniotic endotoxin and interleukin 1 (IL-1) induce lung inflammation followed by lung maturation.
**Table:** 

(a)	C0014264:Endotoxins (Lipid)-causes-C0032285:Pneumonia (Disease or Syndrome)
(b)	C0021755:Interleukin-1 (Amino Acid, Peptide, or Protein)- causes-C0032285:Pneumonia (Disease or Syndrome)

Unlike other systems or models that are limited to extracting one or few specific types of relations, such as drug-drug interactions [25] or chemical-disease relationships [26], SemRep attempts to cover a set of 30 relations and their negated forms based on the UMLS Semantic Network [19]. In a comprehensive evaluation, SemRep was found to yield a precision of 0.55, recall of 0.34, and F_1_ score 0.42 in strict evaluation, and 0.69 precision, 0.42 recall, and 0.52 F_1_ score in relaxed evaluation, which more accurately characterizes SemRep performance [12]. An additional evaluation on a standard benchmark dataset containing chemical-disease relationships [26] yielded a precision of 0.90, recall of 0.24, and an F_1_ score of 0.38 [12]. These evaluations show reasonable to good precision but low recall, revealing a common issue in rule-based systems.

### 2.2. Supervised learning for biomedical RE

Recent studies have shown the effectiveness of fine-tuning Transformer-based large language models for biomedical RE [10,27]. These studies often formulate RE as an entity pair classification task, and use BERT variants, e.g., BioBERT [28] or PubMedBERT [21], pre-trained on biomedical literature to incorporate biomedical domain knowledge. The models are often trained on corpora that focus on a small number of relation types [25,26,29]. Recent corpora includes BioRED [10], in which six entity and eight relation types are annotated, although some relation types are rare. Using this corpus, Lai et al. [27] have shown that combining and aligning several RE datasets could improve model performance, particularly for the rare labels. To perform relation extraction, one can adopt a pipeline approach [15,30,31], performing named entity recognition (NER) first followed by RE, or a joint learning approach learning both tasks jointly [32–34].

In addition to formulating the problem as a relation classification task, generative models have also been recently applied to the RE task. For example, Cabot and Navigli [35] used an autoregressive sequence-to-sequence model, BART-large [36], and pre-trained it on a distantly supervised dataset with more than 200 entity relations constructed from Wikipedia abstracts. The input was structured as a text sequence containing entities and the verbalized subject-relation-object triple, separated by the <sep> token. In the biomedical domain, a study compared the performance of fine-tuning an encoder–decoder transformer (e.g., T5) to that of an encoder-only model (e.g., BioBERT) on ten biomedical benchmark datasets [37]. For T5, the input sequence consists of the input sentences decorated with entity markers to indicate the entity positions followed by the gold label. Similar to Lai et al. [27], they also explored a multi-task learning approach for the T5 model by mixing benchmark datasets and using a task-specific token (the name of the dataset). The results showed that this approach achieved the best results on five out of ten benchmarks. More recently, generative large language models (LLMs) such as GPT-3, have also been applied to biomedical RE task, although the evidence on their utility for biomedical information extraction tasks broadly remains inconclusive [38–42]; while some report competitive results [41], in other studies, these models underperform fine-tuning [38–40,42].

## 3. Methods and materials

### 3.1. Dataset construction

**Fig. 1.**
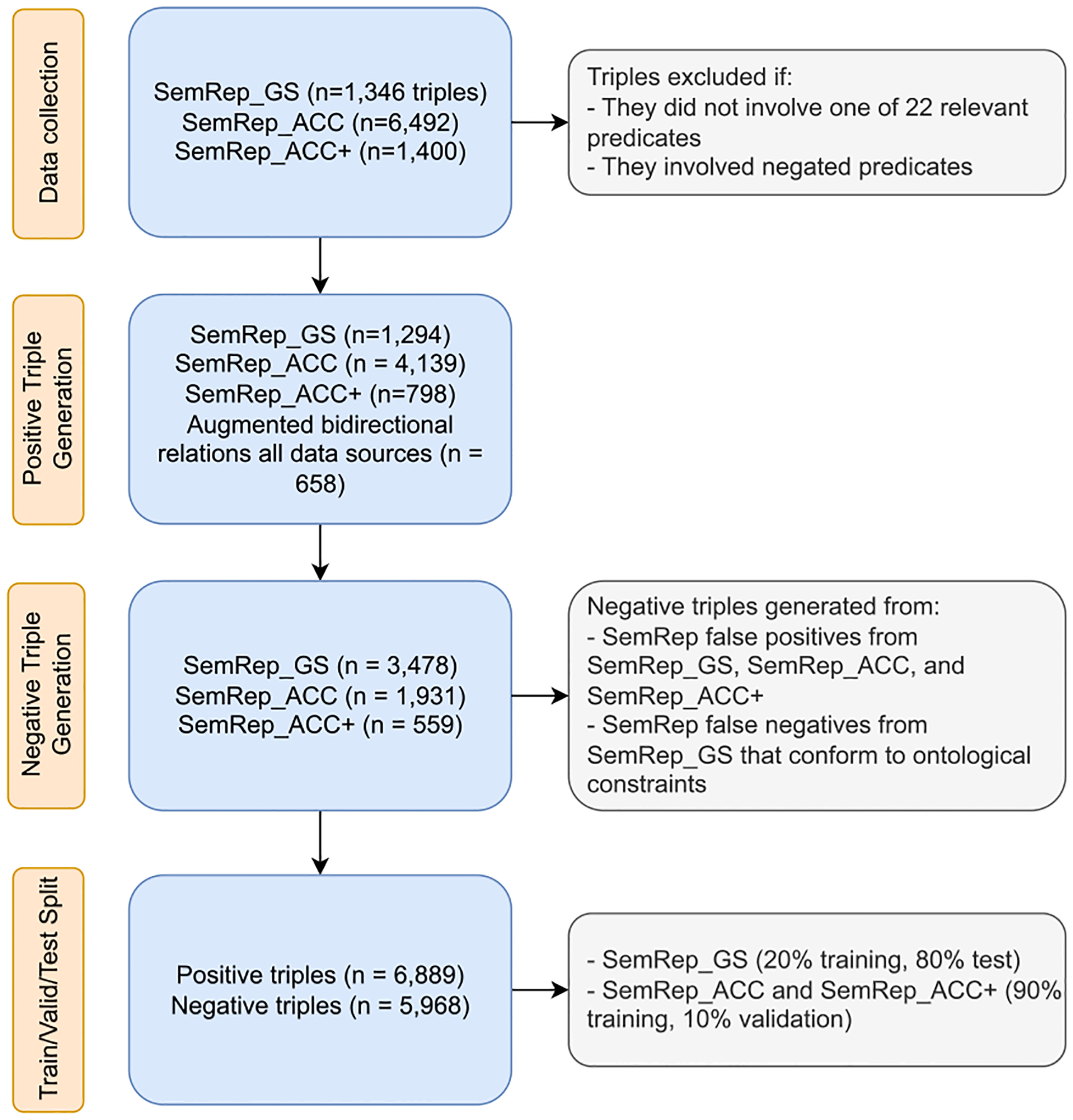
Dataset construction pipeline.

The dataset construction pipeline is illustrated in Fig. 1. Our data collection involved three sources. First, we used the SemRep Gold Standard dataset [19], in which 500 sentences randomly selected from PubMed abstracts were annotated by experts for all relevant semantic triples encompassing 26 relation types and 1,346 triples. We refer to this data as semrep_gs. The second data source is a study in which 6492 triples sampled from SemMedDB [14] were labeled for accuracy by two annotators [6,20]. We refer to this data as semrep_acc. In this study, we additionally annotated the accuracy of semantic triples from a randomly selected set of 1400 triples. These triples involved rare relation types for which there were insufficient number of examples in semrep_gs and semrep_acc. We refer to this dataset as semrep_acc+.

While SemRep is able to generate 30 relation types and their negated forms, not all are represented in semrep_gs in sufficient numbers. For example, while it contains the comparative predicate compared_with, its subtypes, namely higher_than, lower_than, and same_as, are not included, as well as the predicate type complicates. Furthermore, the predicates converts_to, manifestation_of, occurs_in, and method_of, are rare in semrep_gs (fewer than 6 samples). There are also only a total of 30 instances that involve negated predicates. Due to these limitations, we excluded the relation types mentioned above as well as negated forms and focused on the remaining 22 positive relation types in this study, namely: administered_to, affects, associated_with, augments, causes, coexists_with, compared_with, diagnoses, disrupts, inhibits, interacts_with, isa, location_of, part_of, precedes, predisposes, prevents, process_of, produces, stimulates, treats, uses. We refer the reader to Kilicoglu et al. [19] for definitions and examples for these relation types.

Two relation types in SemRep (associated_with, interacts_with) are bidirectional; i.e., their subject and object arguments can be switched, without changing the meaning (e.g., A-associated_with-B is equivalent to B-associated_with-A). Furthermore, for evaluation purposes, the relation location_of is considered inverse of the relation part_of (A-location_of-B is equivalent to B-part_of-A) [19]. For each instance with one of these relation types, we added another instance with reversed arguments due to this bidirectionality. Because interacts_with was already highly represented in the dataset, we only reversed 10% of the interacts_with instances.

The previous steps outlined yield positive semantic triples. To enable model training, negative samples are also required. We generated negative examples from semrep_gs. From the 500 sentences in this dataset, we selected relations from SemMedDB that were not included in the semrep_gs. These correspond to SemRep false positives and can be considered challenging negative examples, as they conform to specific semantic and syntactic constraints used by SemRep. Another set of negative examples (also SemRep false positives) came from semrep_acc and semrep_acc+ datasets. We generated additional negative examples by considering all entity pairs in sentences in semrep_gs that were neither annotated nor false positives, but conformed to ontological constraints followed by SemRep. The negative samples are labeled as no_rel.

We split the data from three sources in different proportions to construct the training, validation, and test sets. Because semrep_gs is the only fully annotated dataset, we set aside most of it (80% of the sentences) for testing and only used 20% of it for training. We used semrep_acc and semrep_acc+ sentences for training and validation only, because they only evaluate triples generated by SemRep and, thus, cannot be used to evaluate recall. We split both datasets into 90% for training and 10% for validation.

### 3.2. Model

#### 3.2.1. The core architecture

**Fig. 2.**
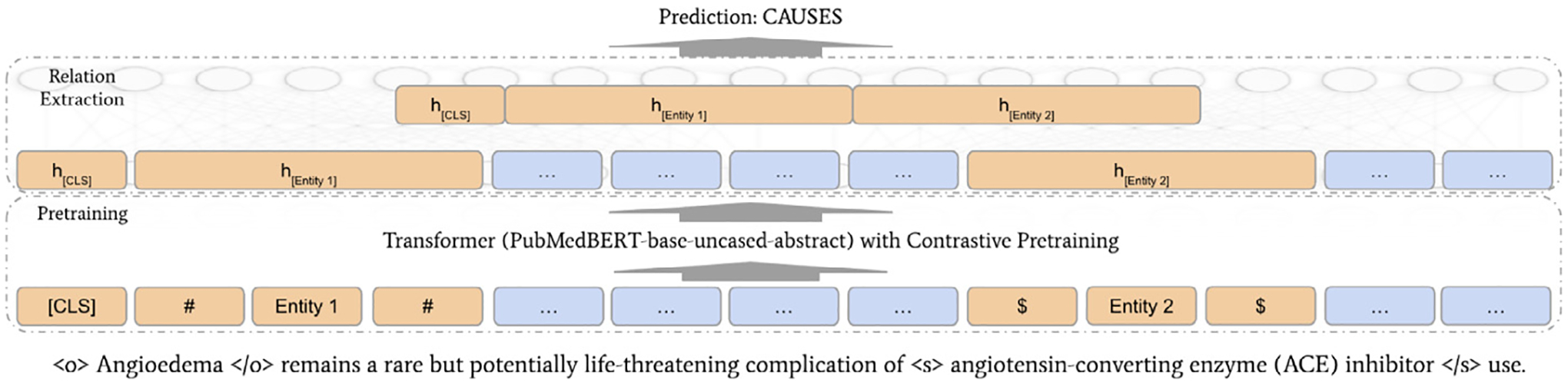
Illustration of the model architecture. It is worth noting that the PubMedBERT was further pre-trained using contrastive learning.

The proposed model aims to predict whether there is a semantic relation between a given pair of entities in a sentence and, if so, predicts its type. Specifically, given an input sequence and the subject and object entities enclosed by 〈*s*〉 and 〈*o*〉 respectively, the model predicts the relation type from the set of 23 pre-defined labels (SemRep relation types and no_rel). The model comprises a contrastive pre-training step followed by a fine-tuning step on our dataset. The model uses the pre-trained PubMedBERT model [21] (*PubMedBERT-base-uncased-abstract*) as its foundation. The core model architecture is illustrated in Fig. 2. We marked the input sentence with entity markers to indicate the positions of the two entities.

Inspired by Su et al. [17], we first applied contrastive pre-training to enhance and generalize the BERT model’s representation to improve the performance of the biomedical relation extraction task. We followed their work by continuously pre-training PubMedBERT using contrastive learning in a self-supervised manner with augmented data. Differently from their work, we employed UMLS-EDA [43], which has been proven to better serve biomedical NLP tasks by incorporating knowledge from the Unified Medical Language System (UMLS) [13]. For each training sample, we generated two samples (positive pairs) that are semantically similar. This augmented data was used as input for continuous pre-training with a batch size of 16.

We followed the implementation of the contrastive learning loss in Su et al. [17]. A feedforward layer with a dropout rate of 0.1 and a Tanh activation function was added on top of the outputs from the last hidden layer of PubMedBERT to enhance representation quality. The loss for a given positive pair *z*′ and *z*″ was calculated as follows. Here, sim represents the cosine similarity between the two samples.


$$\text{loss}\left(z^{\prime },z^{\prime \prime }\right)=-\text{log}\frac{\text{exp}\left(\text{sim}\left(z^{\prime },z^{\prime \prime }\right)/\tau \right)}{\sum_{k=1}^{2N}1_{\left[z_{k}\neq z^{\prime }\right]}\;\text{exp}\left(\text{sim}\left(z^{\prime },z_{k}\right)/\tau \right)}$$

Two samples that did not originate from the same source were paired as negatives. The temperature parameter, denoted as *τ*, was set to 0.1. During pre-training, the contrastive loss of positive pairs is minimized in each batch to maximize their similarity. Simultaneously, the similarity between positive and negative instances is minimized.

After pre-training, we further fine-tuned the model on our training data, adapting the approach proposed by Wu and He [22]. The input for the label classification layer consists of the representation of the entity pair and sentence representation. Entity representation was obtained from the last hidden layer of the sequence output from the PubMedBERT model with continuous pre-training. We then used a mean-pooling operation to obtain embeddings for each entity. The [cls] token and the entity information (including the entity position markers) were independently processed through a feedforward layer with a dropout operation with a rate of 0.1, followed by a Tanh activation function and a layer normalization operation. Next, we concatenate the three resulting vectors: the representations of two entities and the sentence representation ([cls]). We then pass this concatenated vector through a label classifier to obtain prediction scores for each label.

Due to the limited number of samples in certain classes, we weighted each class based on the ratio of samples in that class to the total number of samples in the batch. During the model training process, we employed cross-entropy loss, incorporating these specified class weights. This approach gives more weight to categories with fewer samples, mitigating class imbalance issues.

For each class, we applied different thresholds to the prediction probabilities after applying the softmax function to increase the model confidence in the predictions. Specifically, we used the model predictions on the validation data to calculate the threshold for each relation type that maximized the F_1_ score on the validation set. In a multi-class setting, we used the one-vs-rest mechanism to obtain optimal thresholds for each type. If the optimal threshold is below 0.5, we used 0.5 as the threshold. During testing, if none of the class probabilities exceeded the threshold, the model assigned the no_rel class to the instance.

#### 3.2.2. Use of semantic types and groups

In SemRep, the identification of relations between entities is based on a set of semantic constraints among semantic types, extended from the UMLS Semantic Network [12]. For example, the semantic constraint Amino Acid, Peptide, or Protein-causes-Disease or Syndrome licenses the relation Interleukin-10-causes-Pneumonia in Example 2b. The UMLS semantic types are further aggregated into coarse-grained semantic groups (e.g., Disease or Syndrome is in Disorders group) [44]. Therefore, we explored using semantic types and groups instead of entity names as input to encourage the model to capture higher-level semantic relationships between entity mentions. Specifically, we replaced the entity mentions with their corresponding semantic types and groups to generate two additional sets of data for model training and evaluation. For example, the sentence *“Western blotting analysis indicated that quercetin induces the G0/G1 phase arrest via decreasing the levels of CDK2, cyclins E, and D proteins”*, was changed to *“Western blotting analysis indicated that Pharmacologic Substance induces the G0/G1 phase arrest via decreasing the levels of Gene or Genome, cyclins E, and D proteins”* using semantic types as input and *“Western blotting analysis indicated that Chemicals and Drugs induces the G0/G1 phase arrest via decreasing the levels of Genes and Molecular Sequences, cyclins E, and D proteins”* using semantic groups. To investigate the effects of entity representation, three versions of the training data were created: entity mentions (baseline), entity mentions substituted with semantic types (baseline+semtype), and entity mentions substituted with semantic groups (baseline semgroup).

#### 3.2.3. Experimental settings

The hyperparameters used are as follows: the number of epochs for both pre-training and training processes was set to 10, the batch size was set to 16, and the maximum sequence length was chosen as 384 to reduce computation by eliminating padding space since no samples exceeded this token limit in our dataset. For optimization, we employed the Adam optimizer with a learning rate of 5 × 10^−5^, and the epsilon value for the Adam optimizer was set to 1 × 10^−8^ to enhance training stability.

### 3.3. Evaluation

To evaluate the model’s performance on test data, we employed standard RE evaluation metrics, precision, recall, and F_1_ score. We used both micro- and macro-averaging of these metrics. We exclude the type no_rel from these evaluations. We also calculated Area Under Receiver Operating Curve (AUC) to assess the model’s ability to distinguish between classes (including no_rel) and the statistical significance of the differences between performances of different models using McNemar’s test.

#### 3.3.1. Large-scale assessment

The primary objective of this paper is to enhance SemRep recall and potentially expand the coverage of relations in SemMedDB by incorporating newly discovered relations. To estimate the magnitude of this expansion through the use of the model, we further evaluated our best-performing model at a larger scale using sentences from SemMedDB [45]. Across all years from 2000 to 2023, we sampled 500 abstracts from each year, resulting in a total of 12,000 abstracts. We selected abstracts such that they do not overlap with abstracts in the datasets used for this study (semrep_gs, semrep_acc, and semrep_acc+).

We obtained abstract, sentence, entity, and relation information from SemMedDB version 43 (semmedver43_r) (through September 1, 2023). Because the best-performing model was baseline+semtype (see Section 4.3), we replaced the entity mentions with their corresponding semantic types. Because an entity can have multiple semantic types (e.g., *rabeprazole* is both Organic Chemical and Pharmacologic Substance), we ran the model with all possible pairs of semantic types, selecting the label with the highest probability. For example, in cases where the subject candidate has 2 semantic types and the object candidate has 3 semantic types, we generated 6 input samples from this pair of entities. Besides, given two entities A and B, we considered two distinct samples A-B and B-A, because most relations are unidirectional. We did further pre-processing to filter out subject–object pairs that do not conform to semantic constraints used in SemRep. This process yielded 1,533,716 samples from selected abstracts.

After obtaining model predictions, we applied the same threshold to the predicted label probabilities as we did for the test set in each category. We further verified isa relations by checking the UMLS concept hierarchy, a step that is also performed by SemRep. We compared the resulting predictions to the relations identified by SemRep. To estimate the potential enhancement of SemMedDB coverage with additional relations discovered by the model, we randomly sampled 506 relations that were not previously identified by SemRep (23 samples for each category) and assessed their accuracy.

## 4. Results

### 4.1. Dataset statistics

The resulting dataset consists of 8,255, 761, and 3,841 samples in the training, validation, and test sets, respectively, for a total of 12,857 instances. The label distribution is provided in Table A.1. 53.5% of the instances are positive; interacts_with has the highest number of training and validation examples (1030 and 109, respectively), while process_of has the most test examples (191).

### 4.2. Model comparison

The comparison of models using various input representations with and without contrastive pre-training and SemRep are shown in Table 2. All models outperform SemRep in recall and F_1_ score, while SemRep has higher precision. The best results were achieved with the model that uses semantic types for entity representation and contrastive pre-training (baseline+semtype+contrastive) (0.57 precision, 0.84 recall, 0.68 F_1_ score, micro-averaging). Using optimal thresholds for each relation type learned on the validation set improved the results further by two percentage points in F_1_ (0.62 precision, 0.81 recall, 0.70 F_1_), with increase in precision and decrease in recall, overall effect being positive. Using semantic groups as input entity representation has a slight advantage over simply using the entity mentions; however, its performance remains lower than using semantic types. The improvement of model performance due to contrastive pre-training was limited and evident mostly in macro-averaged results.

The performance differences of all models from SemRep were statistically significant (*p* ≤ 0.001). Differences due to contrastive learning were not statistically significant. Using semantic types had a statistically significant effect over baseline (*p* ≤ 0.05), while using semantic groups did not (*p* = 0.15). Performance difference due to thresholding with the best model was also statistically significant (*p* ≤ 0.001).

### 4.3. Comparison with SemRep

In this section, we compare the performance of the best-performing model (baseline + semtype + contrastive w/ threshold) with that of SemRep in more depth. Results are shown in Table 3. Overall, the model outperforms SemRep significantly (macro-F_1_: 0.62; micro-F_1_: 0.70 vs. 0.39 and 0.46 respectively). As expected, the improvement is largely due to increase in recall, while overall precision remains largely the same. The largest F_1_ score improvement is in relation type administered_to (+0.52), followed by affects (+0.48), predisposes (+0.38) and interacts_with (+0.38). SemRep yielded higher F_1_ score for only one relation type: compared_with (+0.09).

Recall increased for all categories with the model when compared to SemRep, most significantly with associated_with (+0.75), predisposes (+0.73), and interacts_with (+0.70). The model obtained perfect recall for four relation types (location_of, part_of, associated_with, and prevents).

With respect to precision, the results were more mixed. While the model yielded better precision for more than half of the relation types, its precision was lower in others. The highest precision improvement was obtained for affects (+0.32), administered_to (+0.30), and interacts_with (+0.18). Precision degraded most with predisposes (−0.44), prevents (−0.43), and part_of (−0.24). Note that SemRep yielded a precision of 1.0 for predisposes and prevents.

**Fig. 3.**
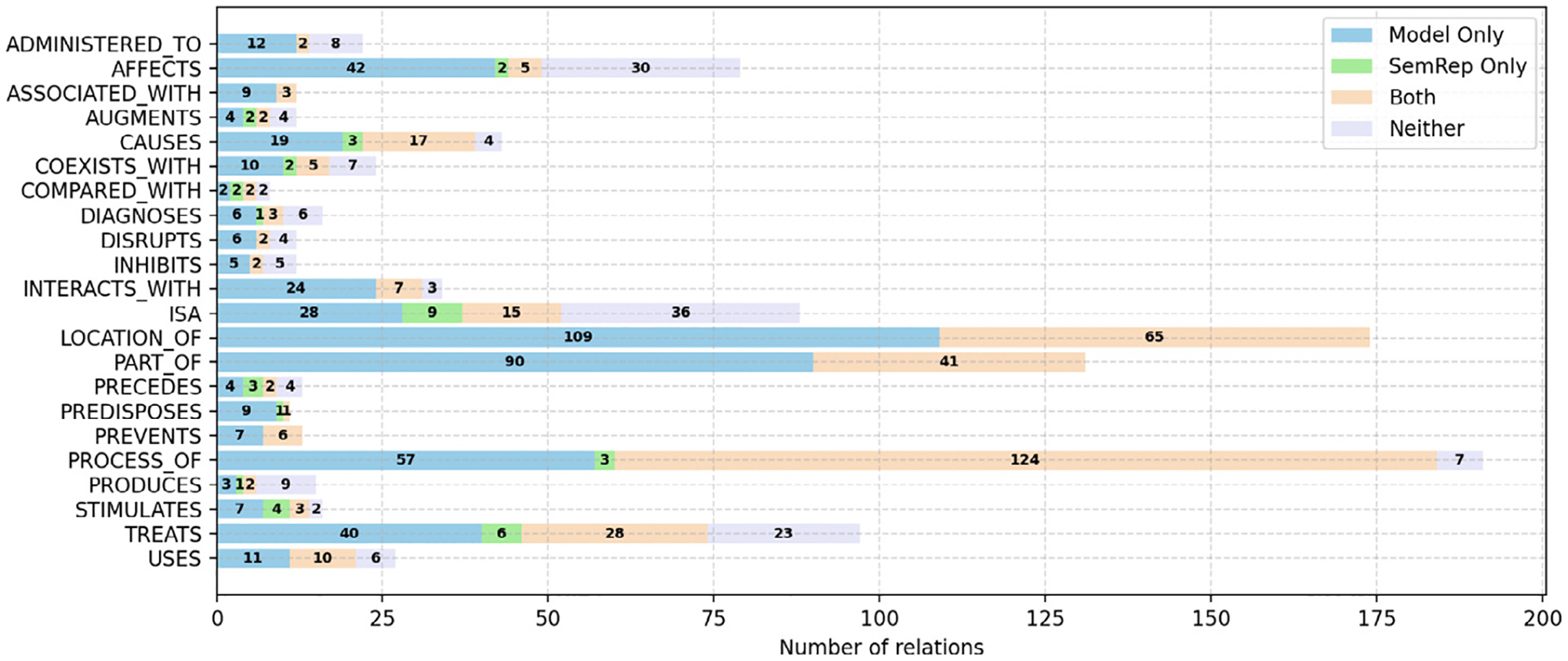
Number of correct relations extracted by SemRep versus the best-performing model for each relation type. Each bar represents a category that is further divided into four stacked bars. Each stacked bar is associated with numbers relative to that category.

One of the major goals of this study was to assess whether a fine-tuned relation classification model could augment SemRep. To that end, we also compared the output of the best-performing fine-tuned model and SemRep. We found that the model correctly identified 504 relations in the test set that were missed by SemRep (~ 59% of the relations predicted by the model). At the same time, it missed 39 gold relations identified by SemRep (~ 10% of the relations extracted by SemRep). The distribution of the overlaps and differences for relation types are shown in Fig. 3.

When examining the correct predictions for each category, detailed in Fig. 3, we find that all instances of some relation types such as prevents, predisposes, part_of, location_of and associated_with are identified by either method or both. isa is the relation type that is missed most by either method in absolute number, while produces is the most missed relation type proportionally.

### 4.4. Large-scale assessment

In this section, we present the results of the best-performing model (baseline + semtype + contrastive w/ threshold) on the 12K abstracts from PubMed, comparing them to the relations found by SemRep and stored in SemMedDB [14].

**Fig. 4.**
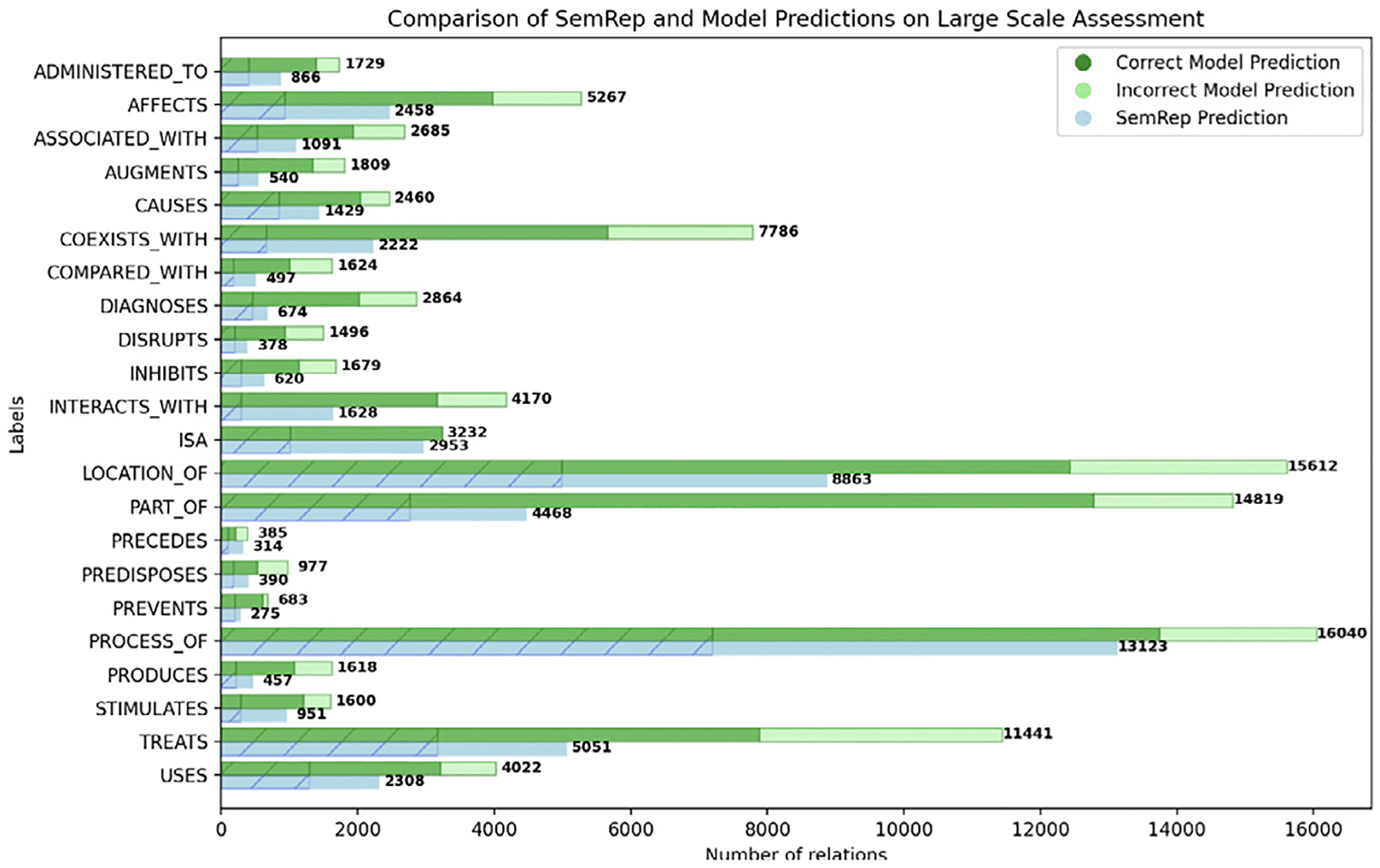
The number of potentially correct relations discovered by the best-performing model compared to SemRep in a large-scale assessment for each relation type. Dark green indicates the magnitude of predicted true positives, while light green indicates the estimated false positives.

The results are presented in Fig. 4. The area with diagonal lines from the top-right to the bottom-left of each bar represents relations found by both SemRep and the model. The model identified a total of 103,999 relations, which is a substantial increase from the 51,564 relations stored in SemMedDB for the 22 relation types. Out of the 103,999 relations, 77,541 were not previously identified by SemRep. SemRep also has 25,242 relations that were not found by the model, highlighted in blue in Fig. 4. The model predicted more relations for each type compared to SemRep. The number of all relation types increased with the model; the largest increase was observed for diagnoses (325%) and the smallest increase for isa (9%).

To assess the quality of these additional relations, we randomly selected and manually evaluated the correctness of 506 predications (23 samples for each 22 category) that SemRep had not previously identified. This process aims to provide a more accurate estimate of potentially correct new relations. 67% of the model predictions were found to be accurate. Assuming this accuracy level is maintained, this would result in an estimated increase in the size of SemMedDB by 51,952 relations for the 12K abstracts considered (~100% increase). Extrapolating this increase to entire PubMed, this would result in about 253M relations for more than 36M abstracts currently in PubMed (the latest SemMedDB release has more than 126M). We estimate the precision for each category based on the small set of human-annotated samples and represent the percentages in the bar chart shown in Fig. 4, which is highlighted in dark green. The light green bar represents the estimate of potential false positive model predictions.

## 5. Discussion

### 5.1. Dataset

By augmenting the relatively small SemRep Gold Standard dataset developed in prior work with data from precision-focused evaluations of SemRep results and negative samples, we were able to generate a moderately-sized dataset that could be used to train and validate relation classification models that can complement SemRep.

### 5.2. Model performance

PubMedBERT-based models significantly outperform the base SemRep. Using semantic types for entity representations improves results over using semantic groups; both representations outperform direct use of entity mentions. By leveraging high-level semantic knowledge, models that use semantic types and groups are trained to capture both syntactic and semantic patterns, rather than relying on the memorization of entity representations, and are therefore likely to be more generalizable. These results also suggest that fine-grained semantic type knowledge may be more helpful than coarse-grained categorizations for identifying biomedical semantic relations, possibly because some semantic groups are somewhat heterogeneous and relations extracted by SemRep are supported by constraints based on semantic types, not semantic groups.

The effect of contrastive pre-training was mostly minor and mainly observed in macro-averaged metrics, which suggests that contrastive pre-training could help improve the model performance for the minority classes. Because we conducted contrastive pre-training using limited data, augmented solely with a single data augmentation method (UMLS-EDA [43]), it could be more effective to explore advanced data augmentation methods or utilize a large-scale, knowledge-intensive pre-training dataset to assess whether contrastive pre-training could yield positive results more consistently.

Comparing the predictions from the best model with SemRep output on the test set, we find that about 59% of the correct model predictions are unique to the model (i.e., not extracted by SemRep). This underscores the potential of the model to discover a significant number of new relations within PubMed abstracts, thus, significantly expanding the coverage of and increasing the utility of SemMedDB [14]. At the same time, we note that 10% of the SemRep relations are unique, indicating that there is some complementarity between the model and SemRep.

To contextualize our results better, we also applied our model, without retraining, to BC5CDR corpus of chemical-induced disease relations [26]. This analysis is presented in Appendix B.

### 5.3. Large-scale assessment

The analysis of the results obtained with the best-performing model on 12K abstracts suggests that the model can double the number of relations included in SemMedDB, significantly enhancing its coverage. This, in turn, could improve the utility of SemMedDB for many downstream tasks for which it has been used, such as literature-based discovery [46], drug repurposing [6], and clinical decision making [47]. In particular, the increased coverage in causal relation types, such as causes, affects, augments, stimulates, and inhibits, with about two-thirds of the predicted relations estimated to be correct, combined with advanced techniques, such as graph neural networks and link prediction, could underpin further mechanistic biomedical insights and discoveries. In some use cases, it might be appropriate to apply SemRep accuracy classifiers [6,20] to the model predictions to obtain a more precise subset at the expense of a small drop in coverage.

### 5.4. Analysis of model predictions

**Fig. 5.**
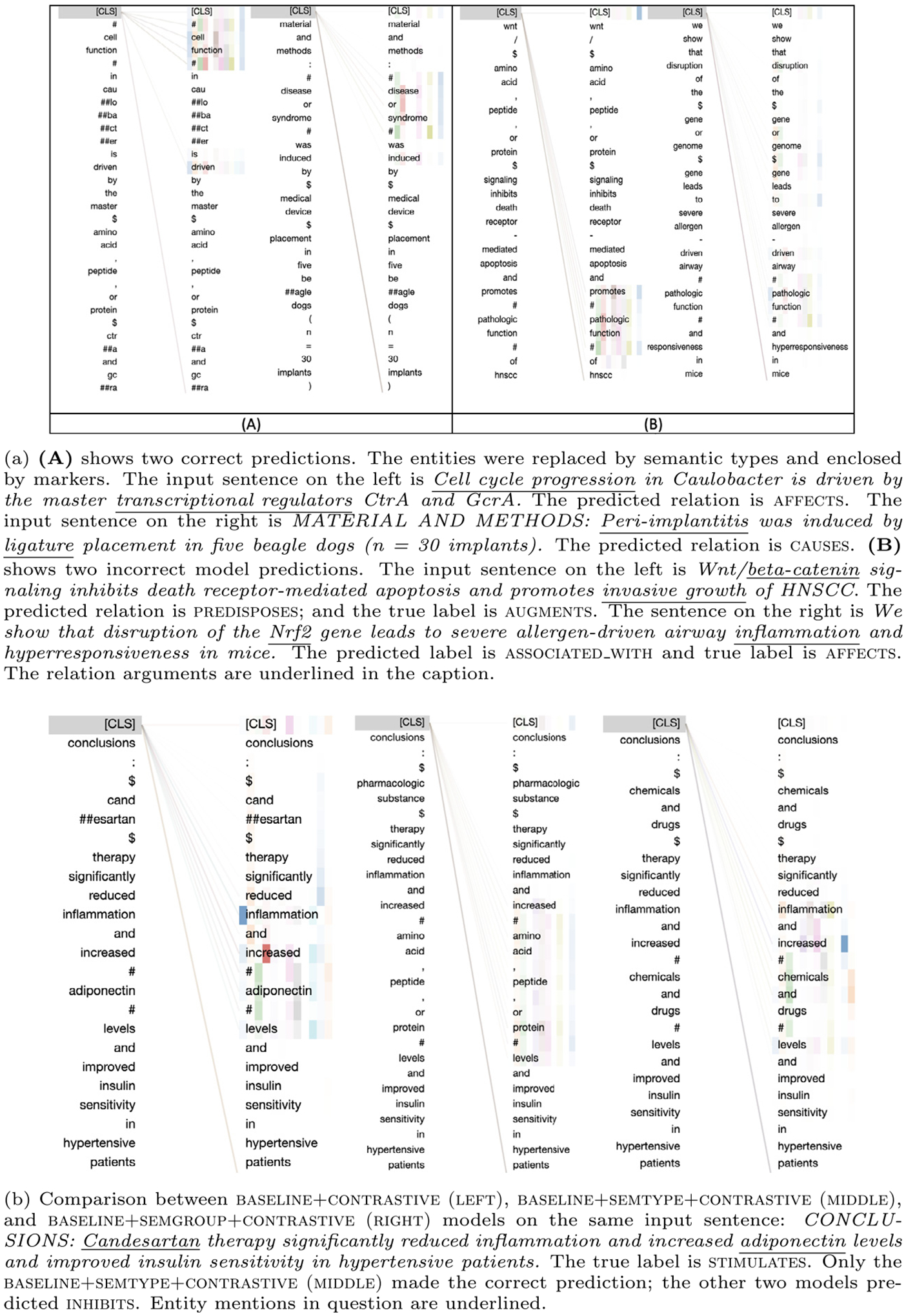
Analysis of model predictions using attention visualization.

To better understand the basis for model’s predictions, we selected both accurate and inaccurate predictions of the best-performing model and visualized attention for the input text. We use the BertViz Python package [48] to visualize attention. Specifically, for each prediction on the test set, we extracted the attention values for each token in the last self-attention layer. These values were normalized across the 12 attention heads and tokens (ranging from 0 to 1). As shown in Fig. 5(a), the weights being visualized are associated with the [cls] token, where the darkest highlighted word is the word with the highest relative importance.

In Fig. 5(a), we visualize attention in both cases (correct and incorrect), revealing how each token influences the model’s final decision. Fig. 5(a) (A) shows two correct examples. For example, on the left, the attention is primarily given to the semantic type of the object argument *Cell cycle progression* (Cell Function) and the trigger *driven* as well as the [cls] token. We note that *driven* is an indicator rule for causal relation types causes, augments, stimulates, but not for affects in SemRep. The emphasis on the [cls] token indicates that the model also pays attention to the overall meaning of the sentence. On the right, we also observe that the attention is mostly on the semantic type Disease or Syndrome and the trigger *induced*, which is also mapped to causes in SemRep. These two examples show that the model captures existing SemRep rules to some extent, while also addressing some potential gaps in these rules.

Fig. 5(a) (B) presents incorrect predictions made by the model. On the left, while the model gives attention to the semantic type (Pathologic Function) and the trigger *promotes*, it predicts as the relation type predisposes instead of the annotated augments. Both the trigger (*promote*) and the semantic type constraints on the arguments in this case are shared between these two relation types augments and predisposes in SemRep rules. Thus, this miscategorization can be attributed to the semantic overlap between these two relation types, which suggests that further ontological differentiation may be needed between some relation types. However, it is still notable that the model implicitly captures the rules explicitly stated in SemRep. On the other hand, in the example on the right, the attention is more scattered between tokens (though mostly still on the arguments and the trigger *leads to* in the final layers). While the fact that there is a relation between the entities is captured, the predicted relation type is a weaker relation (associated_with) rather than the stronger affects relation that was annotated.

We also investigated how models trained with different entity representations (baseline+contrastive, baseline+semtype+contrastive, and baseline+semgroup+contrastive) used attention. An example is provided in Fig. 5(b). The figure shows that when using entity mentions, the model paid more attention to distracting information (*reduced inflammation*) and when using semantic groups, it paid little attention to the [cls] token, seemingly ignoring the overall sentence meaning. In both cases, inhibits is predicted as the relation type. On the other hand, when using the semantic types, most attention is on *increased* and the object argument, as well as the [cls] token, which leads to the correct prediction (stimulates).

We present additional error analysis in Appendix C, focusing on relations that were missed by the model as well as SemRep (as illustrated in Fig. 3).

### 5.5. Limitations of the study

Our study has some limitations. First, because the main focus of the study was on classifying relations between entity pairs, we did not perform NER and relied on concepts recognized by MetaMap. SemRep errors caused by MetaMap concept recognition errors are common [12]. At the same time, SemMedDB contains MetaMap entities for all PubMed articles (about a total of 1.9B entities) and this increases the feasibility of our approach. Improving the NER quality would further enhance the utility of the model and its application to PubMed scale. Second, we excluded some relation types that were not included or existed in only small numbers in the SemRep Gold Standard as well as negated relations. Future work could focus on expanding the dataset with these relation types. Third, our manual evaluation of 506 model-only predictions in large-scale assessment showed that, while technically correct based on the input sentence, some predictions were not sufficiently specific (e.g., treatment treats syndrome). Additional post-processing of the predictions, for example, using SemRep novelty filter [14], could further improve utility of the model predictions.

## 6. Conclusion

In this study, we fine-tuned a PubMedBERT-based model to classify relations between entity pairs in the biomedical literature, augmented by pre-training through contrastive learning. We leveraged the entities already extracted by SemRep (via MetaMap) and assessed whether and to what extent the relation classification approach would complement the SemRep tool and the resource based on it, SemMedDB. Our results demonstrate that relation classification with semantic types can complement SemRep successfully and significantly increase the size of SemMedDB, enhancing its utility for downstream applications.

In future work, we plan to apply the model to the entire SemMedDB dataset and release the predictions to the research community. Improving the quality and specificity of named entities provided as input to the model, extending the dataset to include rare labels that were excluded in this study, and using data augmentation approaches to address these rare labels are other potential future directions.

## Figures and Tables

**Table 1:** Statement of significance.

Summary	Description
Problem	Relation extraction is an essential task in biomedical literature mining, with significant benefits for various downstream applications, such as database curation, drug repurposing, and clinical decision making. Most current biomedical extraction systems are limited in the kinds of relations they address and suffer from low accuracy.
What is already known?	SemRep is a broad-coverage biomedical relation extraction tool that also supports SemMedDB, a widely used, PubMed-scale repository of semantic relations. However, SemRep has low recall, which limits its utility.
What this Paper Adds?	We enhance SemRep coverage using a relation classification approach that leverages entities identified by SemRep. In evaluation, this approach increases SemRep recall significantly (44 absolute percentage points). We also show that this approach complements SemRep and estimate that it can double the size of SemMedDB, increasing its utility for downstream applications.

**Table 2:** Comparison of baseline, baseline + semtype, and baseline + semgroup models with and without contrastive learning. SemRep results on the test set are also included. The micro- and macro-averaged F_1_ score calculation excludes the no_rel label. Results with optimum threshold is only shown for the best-performing model (baseline + semtype + contrastive).

	Macro-averaging			Micro-averaging			
Experiments	Precision	Recall	F_1_	Precision	Recall	F_1_	AUC
semrep	**0.58**	0.31	0.39	0.61	0.37	0.46	–
baseline	0.47	0.72	0.56	0.52	0.80	0.63	0.94
baseline + contrastive	0.49	0.73	0.57	0.53	0.80	0.63	0.94
baseline + semtype	0.51	0.74	0.58	**0.57**	**0.84**	**0.68**	**0.96**
baseline + semtype + contrastive	**0.52**	0.75	**0.60**	**0.57**	**0.84**	**0.68**	**0.96**
baseline + semgroup	0.46	**0.77**	0.56	0.51	**0.84**	0.64	0.95
baseline + semgroup + contrastive	0.49	0.74	0.57	0.54	0.83	0.66	0.95
baseline + semtype+ contrastive w/ threshold	**0.58**	0.71	**0.62**	**0.62**	0.81	**0.70**	0.95

**Table 3:** Results of the best model with thresholding (baseline+semtype+contrastive w/threshold) versus SemRep performance on test set. no_rel label is excluded when calculating aggregate metrics.

	SemRep			baseline + semtype + contrastive w/ threshold			Threshold
	Precision	Recall	F_1_	Precision	Recall	F_1_	
administered_to	0.40	0.09	0.15	0.70	0.64	0.67	0.70
affects	0.32	0.09	0.14	0.64	0.59	0.62	0.79
associated_with	0.38	0.25	0.30	0.48	1.00	0.65	0.85
augments	0.57	0.33	0.42	0.55	0.50	0.52	0.76
causes	0.77	0.47	0.58	0.69	0.84	0.76	0.80
coexists_with	0.19	0.29	0.23	0.31	0.63	0.42	0.50
compared_with	0.57	0.50	0.53	0.40	0.50	0.44	0.55
diagnoses	0.67	0.25	0.36	0.69	0.56	0.62	0.83
disrupts	0.40	0.17	0.24	0.44	0.67	0.53	0.81
inhibits	0.40	0.17	0.24	0.47	0.58	0.52	0.79
interacts_with	0.30	0.21	0.25	0.48	0.91	0.63	0.50
isa	0.80	0.27	0.41	0.65	0.49	0.56	0.74
location_of	0.52	0.37	0.43	0.60	1.00	0.75	0.76
part_of	0.76	0.31	0.44	0.52	1.00	0.68	0.76
precedes	1.00	0.38	0.56	1.00	0.46	0.63	0.82
predisposes	1.00	0.18	0.31	0.56	0.91	0.69	0.50
prevents	1.00	0.46	0.63	0.57	1.00	0.72	0.67
process_of	0.82	0.66	0.74	0.86	0.95	0.90	0.68
produces	0.38	0.20	0.26	0.42	0.33	0.37	0.50
stimulates	0.50	0.44	0.47	0.48	0.63	0.54	0.51
treats	0.55	0.35	0.43	0.71	0.70	0.70	0.50
uses	0.56	0.37	0.44	0.62	0.78	0.69	0.70
no_rel	0.80	0.92	0.85	0.93	0.82	0.88	0.50
Macro-averaging	0.58	0.31	0.39	0.58	0.71	0.62	
Micro-averaging	0.61	0.37	0.46	0.62	0.81	0.70	

## Data Availability

Data and code related to this study are available at https://github.com/Michelle-Mings/SemRep_RelationClassification.

## Appendix A. Dataset statistics

The number of relation types in the dataset is provided in Table A.1.

**Table A.1:** Number of samples for each relation type in training, validation, and test sets.

	Train	Validation	Test	Total
administered_to	189	14	22	225
affects	118	8	79	205
associated_with	278	10	12	300
augments	91	11	12	114
causes	165	11	43	219
coexists_with	290	25	24	339
compared_with	74	11	8	93
diagnoses	155	14	16	185
disrupts	105	9	12	126
inhibits	311	24	12	347
interacts_with	1030	109	34	1173
isa	108	13	88	209
location_of	225	7	174	406
part_of	266	10	131	407
precedes	98	12	13	123
predisposes	88	17	11	116
prevents	99	10	13	122
process_of	666	72	191	929
produces	113	13	15	141
stimulates	232	33	16	281
treats	363	35	97	495
uses	277	30	27	334
no_rel	2914	263	2791	5968
Total	8255	761	3841	12,857

## Appendix B. Model performance on the BC5CDR corpus

We evaluated our best-performing model on the BC5CDR corpus [26], which includes chemical-induced disease relations annotated at the document level. Since our model was trained at the sentence level, we identified sentence-level relations from document-level relations, by retaining only those involving entities that co-occur within a single sentence. Given that several entity mentions can be mapped to the same entity ID, we used all instances containing a pair of chemical and disease entities in a sentence and aggregated them at the document level. To align entity and relation types with BC5CDR annotations, we mapped their entity types (Chemical and Disease) to Organic Chemical and Disease or Syndrome semantic types used in our model.

For the evaluation, following the approach reported for prior SemRep evaluation on BC5CDR [12], any entity pair predicted to have a causal relation type (i.e., causes, affects, augments, stimulates, predisposes, or associated_with) is considered a true positive. Negative samples are generated with chemical-disease entity pairs that are not annotated in the BC5CDR corpus. If any of these negative samples are predicted to have one of the causal relation types, this is considered a false positive.

Out of the 1066 total document-level relations, 740 relations (69%) involve entities that co-occur in a single sentence and are considered. Our best-performing model achieved 0.51 precision, 0.78 recall, and 0.62 F_1_ score for the positive class (0.71 micro-F_1_). In comparison to previous SemRep evaluation on BC5CDR [12], this is significantly higher recall (0.78 vs. 0.35) at the expense of lower precision (0.51 vs. 0.90). This result further suggests that our model can serve as a valuable supplement to SemRep, enhancing the coverage of relations mentioned in biomedical literature. We note that the best relation extraction performance reported on BC5CDR, to our knowledge, is 0.79 F_1_ [50]. However, note that our results are not directly comparable to theirs, because we have not retrained our model on BC5CDR and their models also consider relations that occur only at the document level.

## Appendix C. Error analysis

We analyzed the instances which were missed by both SemRep and the model to better understand the challenging cases. We found that most of these examples (80%) were labeled as no_rel by the model. More than half (56%) were triples with one of the following relation types: isa, affects, and treats.

The isa relation was missed most often, which is partly due to the fact that additional ontological constraints are observed in the extraction of isa relations [11] along with the model prediction. For example, in the example below, the relation between *steroids* and *interventions* is missed because a taxonomic relation does not exist between the corresponding concepts in the UMLS.

1. *The* *interventions* *investigated were: antibiotics (4); home versus hospital administration of antibiotics (1);* *steroids* *(1); mucolytic therapies (6); exercise (3) and pancreatic enzymes (1)*.

Complex sentence structures seem to have led to some false negatives with the relation type treats. An example is shown below, which resulted in six false negatives that involve *TZDs* as the subject.

1. *Other, as yet, unapproved uses of TZDs include:* *non-alcoholic fatty liver disease**, in which TZDs reduced* *hepatic fat accumulation* *and improved liver function tests;* *polycystic ovary syndrome**, where TZDs improved ovulation*, *hirsutism* *and* *endothelial dysfunction**; and* *lipodystrophies**, where TZDs increased body fat (marginally) and decrease liver size*.

Triples with affects relation type were predicted more commonly as another relation type, which seems to be partly due to more complex triggers that the model is unable to recognize. An example is provided below, where the model seems to pay more attention to *decline* instead of *slowed the decline*, classifying the relation as disrupts instead of affects:

1. *The* *high-dose treatment* *slowed a decline of* *motor functions* *of the ALS patients in total Norris and limb Norris scales, but not in bulbar Norris or vital capacity*.
